# Deciphering the developmental trajectory of tissue-resident Foxp3^+^ regulatory T cells

**DOI:** 10.3389/fimmu.2024.1331846

**Published:** 2024-03-28

**Authors:** Fernando Alvarez, Zhiyang Liu, Alexandre Bay, Ciriaco A. Piccirillo

**Affiliations:** ^1^ Department of Microbiology and Immunology, McGill University, Montréal, QC, Canada; ^2^ Infectious Diseases and Immunology in Global Health Program, The Research Institute of the McGill University Health Centre (RI-MUHC), Montréal, QC, Canada; ^3^ Centre of Excellence in Translational Immunology (CETI), Montréal, QC, Canada

**Keywords:** Foxp3 + eT_REG_ cells, transcriptional adaptation, tissue residency, polarization, inflammation, TREG development, mucosal immunity

## Abstract

Foxp3^+^ T_REG_ cells have been at the focus of intense investigation for their recognized roles in preventing autoimmunity, facilitating tissue recuperation following injury, and orchestrating a tolerance to innocuous non-self-antigens. To perform these critical tasks, T_REG_ cells undergo deep epigenetic, transcriptional, and post-transcriptional changes that allow them to adapt to conditions found in tissues both at steady-state and during inflammation. The path leading T_REG_ cells to express these tissue-specialized phenotypes begins during thymic development, and is further driven by epigenetic and transcriptional modifications following TCR engagement and polarizing signals in the periphery. However, this process is highly regulated and requires T_REG_ cells to adopt strategies to avoid losing their regulatory program altogether. Here, we review the origins of tissue-resident T_REG_ cells, from their thymic and peripheral development to the transcriptional regulators involved in their tissue residency program. In addition, we discuss the distinct signalling pathways that engage the inflammatory adaptation of tissue-resident T_REG_ cells, and how they relate to their ability to recognize tissue and pathogen-derived danger signals.

## 1 Introduction

The immune system is capable of both effectively eliminating internal and external dangers and preventing exacerbated immune-mediated tissue pathology. These biological properties, coined disease resistance and disease tolerance, respectively, are complementary and require a controlled balance between pro-inflammatory and regulatory immune responses (1). This is particularly the case in mammalian hosts, where adaptive immunity allows antigen specificity to sustain long-lasting effector and memory responses that can become a potential threat to the function and homeostasis of an affected tissue long after the elimination of the danger. Amongst the mechanisms capable of controlling inflammation-generated pathology, a lymphocyte of thymic origin, a suppressor or regulatory T cell (T_REG_), first described in the late 1960s (2), was shown to be particularly adept at immune suppression. These CD4^+^ T cells express the Forkhead-Box P3 (Foxp3), a lineage-defining transcription factor that governs a large part of their transcriptional program through the repression of pro-inflammatory genes (e.g. *Il2, Ifng*) and the activation of genes essential for their suppressive functions (e.g. *Il2ra* (CD25), *Ctla4, Lag3, Entpd1* (CD39), *Nt5a* (CD73), *Il10, Tgfb1, Gzmb*) (3, 4). In addition, some key signature genes are prominently expressed by these cells when compared to conventional T cells, including *Ikzf2* (Helios), *Tnfrsf18* (GITR), *Nrp1* (Neuropilin 1), and *Itgae* (CD103) (5). In their capacity, T_REG_ cells occupy a central position in the immune response, and are required to ensure tolerance to self-antigens (6, 7), innocuous allergens (8, 9), and commensal microflora (10), promote tissue function and regeneration (11), and prevent and control immunopathology (12).

In a mature immune system, T_REG_ cells isolated from tissues encompass a pool of antigen-experienced CD45RA^−^CD69^+^CD45RO^+^ cells that differ in developmental origin, possess unique functions, and display distinct stages of activation (13). A prominent population of T_REG_ cells found in all organs are tissue-resident T_REG_ (TR-T_REG_) cells that differ from effector memory T_REG_ cells (emT_REG_) in that they display higher levels of the alpha E integrin (CD103) (14), lose CCR7 expression, and lose the ability to re-circulate to lymphoid organs (15). Despite the lack of a consensus on the markers to distinguish TR-T_REG_ and emT_REG_ cells in tissues, recent studies have been able to capture the high degree of transcriptional and post-transcriptional modifications that “precursor” TR-T_REG_ cells acquire to localize to non-lymphoid organs, survive, and adjust their specialized functions *in situ* amidst unfavorable inflammatory, osmotic, or metabolic conditions (16). This program involves the expression of a set of core genes that are typically upregulated, albeit at different levels, by TR-T_REG_ isolated from distinct organs, including the expression of the IL-33 receptor ST2 (17), RORα (18, 19), Icos (20, 21) and Gata3 (22–24). Amongst these differentially expressed proteins, ST2 was recently proposed to distinguish TR-T_REG_ from emT_REG_ (17). Moreover, while there is evidence TR-T_REG_ cells seed non-lymphoid organs, such as the lungs, as early as 8 days of life (25, 26), other TR-T_REG_ cells, like visceral adipose tissue T_REG_ (VAT-T_REG_), accumulate progressively with age (27), suggesting a highly dynamic and developmental path that is largely organ-specific. Critically, there is novel evidence on the developmental trajectory that lead TR-T_REG_ cells to fully establish in the tissue. For example, recent evidence highlights how the TCR repertoire is a central determinant of TR-T_REG_ localisation (16, 28).

Currently, much remains to be understood regarding the origin of TR-T_REG_ cells. Can TR-T_REG_ cells be generated from emT_REG_ cells after the resolution of inflammatory events (29), or do they constitute stable and distinct populations of T_REG_ cells? Seeing that T_REG_ cells found in tissues can originate from the thymic selection process (thymic-derived; tT_REG_) or be generated from the induction of *Foxp3* in naïve CD4^+^ T cells in the periphery (peripherally-induced; pT_REG_), can both subsets be considered TR-T_REG_ cells? Thus, a better understanding of the origin, function, and fate of TR-T_REG_ cells is required before we can harness their therapeutic potential.

**Figure 1:**
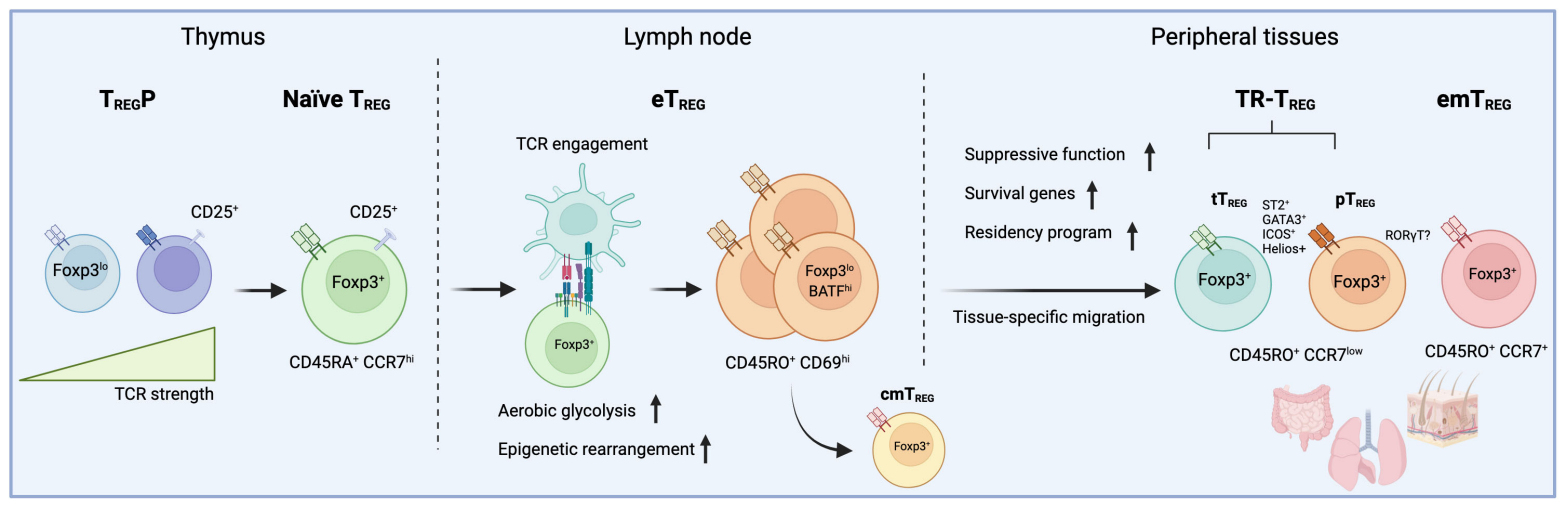
The developmental trajectory of tissue-resident T_REG_ cells involves a series of events starting from thymic selection to peripheral TCR engagement. In this figure, the trajectory of peripheral regulatory T (T_REG_) cells is depicted, as currently defined by recent multi-omics approaches conducted in various lymphoid and non-lymphoid tissues. During thymic selection, precursor regulatory T cells (T_REG_P) expressing self-reactive T-cell receptors (TCR) give rise to a pool of naive CD45RA^+^CCR7^hi^ regulatory T cells (T_REG_). Once in circulation, these T_REG_ cells encounter their specific antigen, triggering an activation cascade that results in a metabolic shift and chromatin remodeling. Subsequently, CD45RO^+^CD69^hi^ effector regulatory T cells (eT_REG_) can either stay in lymph nodes as central memory (cmT_REG_) or migrate to tissues, where they become tissue-resident (TR-T_REG_) or effector memory regulatory T cells (emT_REG_). While thymic-derived TR-T_REG_ cells comprise a large portion of T_REG_ cells in tissues, T_REG_ located in the gut, for example, include peripherally-induced regulatory T cells (pT_REG_). The absence of clear markers poses a challenge in distinguishing between these two populations *in situ*. In addition, while TR-T_REG_ cells isolated from various tissues typically display a conserved phenotype marked by the expression of ICOS, ST2, Helios, and GATA3, a significant portion of T_REG_ cells in the gut exhibit a distinctive RORγT-driven phenotype. Interestingly, there is cumulating evidence that T_REG_ cells lacking Helios expression may be more driven to express RORγT, suggesting a possible segregation between TR-T_REG_ cells derived from the thymus or induced in the periphery.

In this review, we describe the steps required for the generation of TR-T_REG_, starting from thymic selection and spanning to TCR engagement in the periphery, the switch to distinct metabolic strategies, and the modulation of Foxp3 expression that enables the adoption of key epigenetic and transcriptional changes, which, in turn, lead to the expression of a program that is highly adapted to the target tissue (**Figure 1**). These processes involve signaling pathways that can, when in excess, hinder, either temporarily or permanently, the stability of their core transcriptional program, revealing mechanisms by which local inflammation guides the timing and potency of immune suppression. Finally, we attempt to guide the reader through the unique signaling events that can lead tissue-resident T_REG_ cells to control type 1, type 2, and type 3-driven inflammation.

## 2 Origin of tissue-resident T_REG_ cells

Commitment of the T_REG_ cell lineage can occur at various stages of the T cell life cycle. During their development in the thymus, immature thymocytes are selected for the establishment of a functional TCR repertoire. Subsequently, self-reactive thymocytes are either clonally deleted or diverged into a regulatory cell fate as part of a process known as central tolerance. Despite this, a very small fraction of thymocytes escape central tolerance stochastically and must be kept in check by self-reactive thymic-derived T_REG_ cells (tT_REG_), making them critical mediators of peripheral tolerance. Importantly, the events giving rise to tT_REG_ cells require optimal TCR signals and a unique combination of cytokines. However, the peptide pool to which thymocytes are exposed to during this selection process does not ensure complete tolerance towards innocuous non-self-antigens such as commensal bacterial peptides or allergens.

This type of peripheral tolerance often requires the *in situ* induction of peripheral T_REG_ cells (pT_REG_) that possess unique non-self TCR repertoires (30–32) and confer them with non-redundant roles in maintaining homeostatic conditions at barrier sites like the lung and colon. In adoptive transfer models, pT_REG_ cells are capable of suppressing local inflammation in both the colon and the lungs (32–34), but are less efficient at suppressing systemic inflammation (31). Indeed, the distinct transcriptional profiles of tT_REG_ and pT_REG_ cells indicate they favour different suppressive mechanisms that vary in effectiveness in a context-dependent manner (31). Yet, despite these potential differences, attempts at identifying markers in pT_REG_ cells that are distinct from tT_REG_ have so far failed (35, 36), rendering them mostly undistinguishable at barrier sites. While Helios and Neuropilin 1 (Nrp1) are highly expressed by tT_REG_ cells (30, 37), neither Helios (36) nor Nrp1 (38), were found to be exclusively expressed by these cells. Thus, despite their distinct origin, TCR repertoire, and functions, pT_REG_ cells cannot be distinguished from the pool of tT_REG_ cells in mucosal tissues, and further investigation into features that demarcate each subset is warranted.

### 2.1 Thymic development of T_REG_ cells

Thymic-derived T_REG_ cells undergo the same early core processes of thymic selection as conventional CD4^+^ T cells (39, 40). Namely, newly seeded thymocytes undergo V(D)J recombination in the thymic cortex to generate productive TCR chains capable of self-MHC recognition. Upon successful TCR signaling, committed thymocytes migrate into the thymic medulla where they encounter medullary thymic epithelial cells (mTECs) that express promiscuous transcription factors AIRE and Fezf2, allowing them to transcribe and present tissue-restricted antigens (TRAs) to developing thymocytes (41, 42). Here, thymocytes that are strongly reactive toward TRAs and other self-antigens are deleted, while weaker stimulation and the presence of certain cytokines such as TGF-β and IL-2 can skew their fate toward T_REG_ cell differentiation (43–47).

Optimal TCR signaling is the predominant factor driving T_REG_ cell lineage commitment in the thymus. TCR:peptide-MHC interaction triggers a series of phosphorylation events resulting in downstream activation of NFAT, AP-1, and NF-κB family transcription factors (48, 49). Together, these events lead to different T cell lineage specification in the thymus, as well as T cell survival, expansion, and effector function in the periphery. Expression level of the orphan nuclear receptor Nur77 (*Nr4a1*) has been directly linked to TCR signaling strength, and its expression level is elevated in T_REG_ cells compared to conventional T cells in a TCR-dependent manner (50). Unsurprisingly, since co-stimulatory molecules such as CD28 profoundly augment TCR signaling strength via NF-κB activation, they were found to play an essential role in tT_REG_ cell differentiation (46, 51–53). *Foxp3* transcription is intricately regulated by transcription factor complexes binding at its promoter and four conserved noncoding sequences (CNS), termed CNS0 to CNS3. Upon TCR stimulation, downstream activation of the NF-κB pathway results in the recruitment of c-Rel to the *Foxp3* locus at CNS3, which acts as a *Foxp3* transcriptional enhancer that is responsive to TCR signaling alone (54, 55). By dissecting each CNS region through targeted mutations, Zheng and colleagues demonstrated that CNS3 is the region that acts as a pioneer element to the generation of tT_REG_ cells, while CNS1, a region known to bind TGF-β-induced SMAD factors, and CNS2, a region targeted by CREB and STAT5 signals, were not essential to the induction of Foxp3 in tT_REG_ precursors (55), which still require cytokine signaling to become mature and functional tT_REG_ cells (43–47).

Cytokines, particularly common γ (γc) cytokines, are critical for T_REG_ cell development. IL-2 is known to be essential for commitment to the T_REG_ cell lineage (56, 57), as well as its maintenance (58). IL-2 signaling mediates STAT5 binding to the distal enhancer CNS0 as well as the promoter of *Foxp3* (56, 59), and sustains the constitutive expression of Foxp3 through CNS2 binding (57, 59). Not only does STAT5 directly facilitate Foxp3 transcription, Foxp3 also binds to the IL-2 receptor alpha chain (IL2Rα) as a transcriptional activator (60). Completion of this feedforward loop via paracrine IL-2 signaling is obligatory for T_REG_ cell development and homeostasis. Other STAT5-activating γc cytokines have also been linked to T_REG_ cell development, albeit mostly as a compensatory mechanism for impaired IL-2 signaling (45). In addition, TGF-β has also been implicated in tTreg development. While either of its downstream transcription factors, SMAD2 or SMAD3, can directly regulate Foxp3 transcription (61, 62), deletion of the SMAD binding site in the *Foxp3* locus predominantly affects the induction of pT_REG,_ but not tT_REG_ cells (62, 63). Yet, deletion of the TGF-β receptor TβRI during thymocyte development results in severely reduced T_REG_ cell numbers and defective T_REG_ cell function (64). Nonetheless, a recent study might reconcile the paradoxical discoveries. SMAD3/4 can trigger a PKA-dependent signaling cascade that causes the cessation of TCR signaling (65). Thus, the role of TGF-β in tT_REG_ differentiation could most likely be attributed to its effects on TCR signaling rather than direct transcriptional regulation of Foxp3.

### 2.2 The role for thymic selection events in the genesis of tT_REG_ and pT_REG_ cells.

In recent years, accumulating evidence shows that the nature of TCR signaling during thymic selection influences T_REG_ cell response to signals long after thymus egress. Notably, TCR engagement during thymic selection is a critical step in the establishment of a CpG hypomethylation pattern that characterises the epigenetic background of tT_REG_ cells (66). Numerous studies have identified two distinct tT_REG_ precursor (T_REG_P) populations thought to develop into CD25^+^Foxp3^+^ tT_REG_ cells (47, 67–69). The more common CD25^-^ Foxp3^low^ and less abundant CD25^+^ Foxp3^-^ T_REG_P cells were shown to have distinct TCR repertoires with affinity to auto-antigens (67). In the thymus, the two T_REG_P populations display different cytokine and TCR-signaling requirements (47). Importantly, CD25^+^ T_REG_P-derived T_REG_ cells are specifically capable of suppressing experimental autoimmune encephalitis (EAE), whereas Foxp3^low^ T_REG_P-derived T_REG_ cells cannot (67), suggesting a functional bias within the T_REG_ population. For example, murine T_REG_ cells from the colonic lamina propria that express the same TCRα/β sequence have related transcriptional programs (70), illustrating the close relationship between TCR and the transcriptional fate of antigen-experienced memory T_REG_ cells.

Interestingly, while the relationship between TCR specificity and the establishment of TR-T_REG_ cells is not entirely understood, there are experimental examples that suggest the TCR repertoire generated during thymic selection is critical to the destination of both tT_REG_ and naïve T cells. For example, T_REG_ cells transgenic for a VAT-T_REG_-derived TCRα/TCRβ gene arrangement will preferentially migrate to adipose tissue and differentiate into VAT T_REG_ cells (28). Yet, while these observations suggest TR-T_REG_ cells possess a largely self-specific TCR repertoire, earlier work in viral infection mouse models demonstrated that antigen-experienced T_REG_ cells with predominantly non-self TCR repertoires are generated during tissue injury and activate during re-infection (13, 71), suggesting they also contribute to the TR-T_REG_ pool. In addition, in transgenic mice possessing a fixed TCR-β sequence isolated from a Foxp3^+^RORγT^+^ colonic T_REG_ cell, T cells upregulate Foxp3 in the mesenteric lymph node prior to expressing RORγT in the colon (72). As such, both self and non-self-reactive TCR repertoires are key drivers in the generation of TR-T_REG_ cells.

### 2.3 The role of IL-2 and TGF-β

While the strength of TCR signaling acts as the predominant driving force for tT_REG_ cell differentiation, cytokines play a more influential role in the periphery both in maintaining tT_REG_ homeostasis and generating pT_REG_ cells. The signals that lead to the generation of pT_REG_ cells involve chronic suboptimal TCR signaling (73–75) and cytokines such as TGF-β and IL-2 to generate Foxp3-expressing T_REG_ cells *in vitro* (76, 77) and in tissues (78–80). In addition, TGF-β has been shown to strongly promote Foxp3 induction through its downstream transcription factors (SMAD2 or SMAD3) that target CNS1 (61). Consequently, a deletion of CNS1 predominantly affects the induction of pT_REG_, but not tT_REG_ cells (63). Lastly, pT_REG_ cell induction via TGF-β can be further augmented by DC-derived retinoic acid in the lamina propria as well as short chain fatty acid metabolites of commensal bacteria (81, 82), ensuring the establishment of tolerance at mucosal surfaces. While these examples of signals that promote pT_REG_ induction are part of a complex signaling system that merits its own review, they share the common outcome of facilitating *Foxp3* expression in tissue-resident T cells, and further the importance of this transcription factor in forming the regulatory program of tissue-resident CCR7^low^CD69^+^CD45RO^+^ T_REG_ cells.

## 3 The epigenetic and transcriptional trajectory of T_REG_ cells

The factors that regulate the differentiation of TR-T_REG_ remain to be fully understood. Miragaia and colleagues demonstrated through single-cell RNA-seq analysis of lymphoid and non-lymphoid (colon and skin) T_REG_ cells that these tissue-specific adaptations originate from events happening in their respective draining lymph node (19). By tracing TCR clonotypes from draining lymph nodes to their respective tissue, the authors were able to establish a pseudo-space relationship detailing the series of events that drive the generation of specialized T_REG_ cells. They were able to establish that T_REG_ cells are activated, switch to a glycolytic metabolism, and cycle rapidly prior to acquiring genes involved in migration to the tissue (19), revealing conserved stages involved in the generation of TR-T_REG_ cells. As such, this seminal work provided confirmation that progressive transcriptional changes guide the generation of eT_REG_ cells that become TR-T_REG_ cells and highlighted how, despite tissue-specific differences, these cells share a series of epigenetic modifications that allow them to migrate, survive, and function at specific non-lymphoid sites.

### 3.1 The importance of peripheral TCR engagement in the generation of TR-T_REG_ cells

The engagement of the TCR of naïve T_REG_ cells is an important prerequisite for the development of tissue-specialized T_REG_ cells (83, 84), as it promotes a signaling cascade that elicits the expression of key regulatory genes leading to the suppressive activity of T_REG_ cells (85). Additionally, TCR engagement can induce epigenetic and transcriptional changes in T_REG_ cells, some of which are directly influenced by Foxp3, while others act independently (66). People affected by a loss-of-function mutations in STIM1 or ORAI1, proteins involved in store-operated calcium entry (SOCE), encounter a loss of peripheral tolerance despite some cases displaying normal T_REG_ numbers in circulation (86, 87). Similarly, impairing the normal Ca^2+^ influx during TCR engagement by deleting proteins that form the Ca^2+^ release-activated Ca^2+^ (CRAC) channels (STIM1 and STIM2) in mice specifically prevents the differentiation of activated T_REG_ cells into follicular and tissue-resident memory T_REG_ cells and generates a cascade of inflammation leading to multiorgan autoimmunity (88).

### 3.2 Aerobic glycolysis in the activation and clonal expansion of T_REG_ cells

Another critical factor involved in the differentiation and clonal expansion of activated T_REG_ cells is the adoption of aerobic glycolysis. This was notably demonstrated in the skin, as aerobic glycolysis by activated T_REG_ cells is required prior to their migration (89). This may, at first glance, seem counter-intuitive, as there is ample evidence that mature T_REG_ cells adopt fatty-acid oxidation (FAO) as a critical metabolic strategy to survive and suppress immune responses in tissues (90). Yet, while less efficient than oxidative phosphorylation (OXPHOS), adopting aerobic glycolysis is a critical step that occurs during T cell activation by rapidly providing the needed energy for expansion and migration, all-the-while maintaining fatty acid and amino acid reserves for cell division and protein synthesis (91). This is further evidenced by the fact that the mammalian target of rapamycin 1 (mTORC1) which is required for aerobic glycolysis, is not necessary for the thymic or peripheral development of T_REG_ cells, but essential to the function and activation of T_REG_ cells (92). Indeed, to avoid losing their suppressive program, T_REG_ cells balance the intensity of the mTORC1 and mTORC2 pathways (93), a process that is critical during their differentiation. Importantly, however, increasing glycolytic metabolism in T_REG_ cells temporarily deprives them of their suppressive capacity (90, 94), providing further evidence that the differentiation and clonal expansion of T_REG_ cells is contained within a short window of time. As such, the maturational process leading T_REG_ to become eT_REG_ cells requires both TCR engagement and a shift in their metabolic strategy (**Figure 1**).

### 3.3 The role of Foxp3 in the specialization of memory T_REG_ cells

The Foxp3-driven transcriptome of T_REG_ cells is comprised of a T_REG_-specific gene signature and a gene set associated with an activation program which is shared with conventional T cells (95). A lymphoproliferative pathology had been previously observed in “Scurfy” mice where the X-linked *Foxp3* gene encountered a frame-shift mutation that completely disrupts the transcription of Foxp3 (96), confirming the key role of Foxp3 in establishing the suppressive program of T_REG_ cells. Point mutations in *Foxp3* that interfere with its function are the cause of a frequently fatal pediatric hereditary syndrome called immune dysregulation, polyendocrinopathy, enteropathy X-linked (IPEX) syndrome (97), featuring early onset diabetes, severe diarrhea, and eczema, which highly reflects the pathology of ‘Scurfy” mice. Restoring Foxp3 transcription in mice whose T_REG_ cells were genetically engineered to block Foxp3 expression rescues them from severe autoimmunity as it effectively reinstates their suppressive function (12). However, while Foxp3 is essential for the establishment of T_REG_ cells, it does not determine, by itself, the entire epigenetic and transcriptional identity of mature T_REG_ cells (5, 98, 99). Rather, Foxp3 ensures that inflammatory and non-inflammatory signals encountered in the periphery do not destabilise the core suppressive program of T_REG_ cells (98, 100).

Evidence for the unique roles of Foxp3 in non-lymphoid tissues comes from the observation that functional single nucleotide polymorphisms (SNPs) in the human *Foxp3* gene do not generate a homogeneous pathology (97), with multiple accounts of IPEX-related mutations having distinct functional consequences on T_REG_ cells (101). By transposing human-isolated *Foxp3* mutations in conserved murine *Foxp3* motifs, Leon and colleagues confirmed that spontaneous multiorgan autoimmunity is largely attributed to mutations in the DNA-binding motifs, while mutations outside these motifs, notably in the N-terminal regions, lead to organ-specific dysregulation of T_REG_ cell function (101). In particular, a K199del mutation situated in the zing-finger (ZF) domain or mutations R51Q or C168Y in the N-terminal regions are prone to generating symptoms of enteropathy and skin disorders, while a R337Q mutation in the DNA-binding Fork-head domain can, in addition to these symptoms, lead to the development of diabetes mellitus (101). In addition, a murine model mimicking an A384 mutation in *Foxp3* was shown to specifically impair T_REG_ cell function in the periphery, directly impairing the ability of Foxp3 to recognize target genes and altering BATF expression (102), a key transcription factor required for TR-T_REG_ generation (103). As such, the ability of Foxp3 to interact with multiple partners is required to preserve the functional integrity of T_REG_ cells in peripheral tissues.

Although there are elements that suggest protein-protein interactions are critical to this process, we are currently limited in our understanding of how the different molecular complexes that partner with the N-terminal region of Foxp3 (104, 105), such as Tip60, Hdac7, Hdac9, Gata3, c-Rel, Foxp3, Runx1 or Eos, influence the specialization of T_REG_ cells. This is imparted by the fact that it is particularly difficult to dissociate their functions during the early events leading to the differentiation of these cells and the events that happen later in the tissues. One such example is the interaction of Foxp3 with the chromatin remodeling transcription factors TCF1 (encoded by *Tcf7*) and lymphoid enhancer binding factor 1 (Lef1) of the high-mobility group (HMG) family. In mice, the combined knock-out of both *Tcf7* and *Lef1* (*Foxp3* ^CRE^ *Tcf7* ^fl/fl^ *Lef1* ^fl/fl^) does not perturb lymphoid T_REG_ cells but hinders the capacity of colonic T_REG_ cells to suppress DSS-mediated colitis (106). Mechanistically, the molecular complexes TCF1 and Lef1 form with Foxp3 allow T_REG_ cells to control inflammation by repressing genes associated with excessive cycling and cytotoxic function (*GzmB*, *Prf1, Ifng*) and promoting genes associated to a T_REG_ suppressive program (106). Bulk RNAseq of murine mesenteric T_REG_ cells deficient in TCF1 (*Foxp3* ^CRE^ *Tcf7* ^fl/fl^) show enhanced expression of core genes (including *Il2ra*, *Foxp3*, *Tgfb1* and *Lef1*), and a concomitant increase in both pro-inflammatory genes (including *Il6ra*, *Ifngr2*, *Stat3*) and genes involved in TCR activity compared to T_REG_ cells from control mice (107). These data suggest that TCF1 helps maintain a core T_REG_ program and suppress the expression of pro-inflammatory genes during TCR engagement. Similarly, Lef1 is part of an activated T_REG_ program (108), and *in vitro* gain-of-function experiments reveal it reinforces the expression of Foxp3 target genes (108). As such, these observations indicate that when Foxp3 is abundantly expressed, it interacts with both TFs to suppress pro-inflammatory gene expression and reinforce its own transcriptional profile (109). Yet, both murine and human activated (CD45RO^+^) T_REG_ cells display lower *Tcf7* and *Lef1* expression than conventional T cells (T_CONV_) (110) as Foxp3 directly suppresses *Tcf7* transcription and protein production, and reduces chromatin accessibility in regions targeted by TCF1 (95). As such, the highly-regulated chromatin-remodelling effect of TCF1 and Lef1 on T_REG_ cells are likely required for their further differentiation and effector function. Furthermore, pseudo-time analysis from single cell RNA-seq data of lymphoid and non-lymphoid activated T_REG_ cells reveals *Tcf7* and *Lef1* to be particularly expressed by lymphoid T_REG_ cells prior to their tissue migration (19), reinforcing the notion that TCF1 and Lef1 are involved during the early specialization events of T_REG_ cells. For example, a T_REG_-specific depletion of *Lef1* abolishes the generation of follicular T_REG_ (T_FR_) (107), suggesting Lef1 promotes the generation of these cells in a process similar to what is observed in follicular helper T cells (T_FH_) (111). In addition, when compared to murine activated TCF1^-^ T_REG_ cells, TCF1^+^ T_REG_ cells display higher mRNA expression of transcription factors associated to helper T cells, including *Gata3*, *Tbx21* and *Rorc* (107). Collectively, these examples highlight how changes in chromatin accessibility in T_REG_ cells happen mostly after TCR engagement in the lymph node. Nonetheless, Lef1 and TCF1 are but a part of a wide network of known Foxp3-binding partners (104) whose role in defining the specialisation of T_REG_ cells remain ill-defined.

### 3.4 Epigenetic control of T_REG_ differentiation

To effectively reach the tissue, T_REG_ must undergo a series of epigenetic and transcriptional changes that ensure chromatin accessibility in key genes (112). Interestingly, direct comparison between human and murine T_REG_ cells reveal evolutionarily conserved epigenetic mechanisms involved in defining a T_REG_ cell program (110). Histone methylation is an important component in the processes that govern DNA accessibility and, ultimately, a T_REG_ cell transcriptional signature. Importantly, while T_REG_ cells undergo a series of chromatin remodeling events, they actively maintain CpG motif demethylation within the intronic enhancer CNS2 of the *Foxp3* locus (55, 113, 114). Maintaining an open chromatin structure in the CNS2 allows for the robust transcription of *Foxp3* by multi-molecular complexes including Foxp3 itself, NFAT, c-Rel, STAT5, Runx1-CBFβ, CREB/ATFx and Ets1 (114–118). Incidentally, a loss of any of these transcription factors or the methylation of CNS2 impairs the transcription of *Foxp3* and, ultimately, the suppressive function of T_REG_ cells in the periphery (114–119), confirming that Foxp3 is critical for the stability of the transcriptional program of tissue-localised T_REG_ cells.

Tagmentation-based whole-genome bisulfide sequencing of lymph node and tissue-isolated murine T_REG_ reveals these cells undergo multiple rounds of DNA alterations before adopting a tissue-residency program, with up to 4000 genes involved in either gain or loss of methylation (120). The processes that govern the establishment of a T_REG_ program by histone modifications have been elegantly reviewed by Joudi and colleagues (121). Globally, a delicate balance between DNA methyltransferases (DNMTs), ten-eleven translocation dioxygenases (TET), histone acetyltransferases (HATs), and histone deacetylases (HDAC) govern the stability of the T_REG_ cell transcriptional program (119, 121), but can be directly influenced by polarizing signals provided during TCR engagement.

Methylation of cytosines located in CpG-rich regions are largely governed by Dnmt1, Dnmt3a and Dnmt3b (122, 123). Interestingly, the conditional deletion of Dnmt1, but not Dnmt3a, in murine T_REG_ cells causes a loss of peripheral tolerance by 3 to 4 weeks of life, yet the cells maintain their expression of Foxp3 (124). However, these T_REG_ cells display enhanced expression of pro-inflammatory cytokines (IFNγ, IL-6, IL-12, IL-17, IL-22), chemokine receptors (CCR1, CXCR6), and transcription factors (Runx2, Stat3), highlighting the role of Dnmt1 as a non-redundant epigenetic silencer (124). During the S phase, Dnmt1 acts in partnership with the epigenetic regulator ubiquitin-like with plant homeodomain and RING finger domains 1 (Uhrf1) to govern the suppression of these gene loci (125, 126), making both Dnmt1 and Uhrf1 important therapeutical targets for the control of T_REG_ stability and function. Yet, because of the necessity of T_REG_ cells to acquire a set of genes associated with pro-inflammatory T cells, it remains to be understood how both regulators act during T_REG_ cell generation. For example, pharmacological inhibition of PI3K through its PIP4K-associated kinase results in a specific decrease in Uhrf1 in human T_REG_ cells but not TCONV cells (127), suggesting that the strength of TCR signaling plays a role in the way T_REG_ cells govern DNA accessibility of pro-inflammatory genes. In addition, signaling by TGF-β leads to the phosphorylation and subsequent sequestration of Uhrf1 outside the nucleus (128), possibly preventing its partnering with Dnmt1.

On the other hand, the modulation of histone acetylation and deacetylation on the epigenetic adaptation of T_REG_ cells remains ill-defined. Foxp3^+^ T_REG_ cells have been found to express histone acetyltransferases (HAT), including p300, Tip60 and CBP, as well as most members of the histone deacetylase family (HDAC) (129). Pan-HDAC inhibitors, for example, promote the acetylation of *Foxp3* and the suppressive functions of T_REG_ cells (130), confirming the importance of regulating histone acetylation to maintain a T_REG_ transcriptional program. Interestingly, HATs and HDACs are clearly involved in the helper differentiation of T_CONV_ cells (131), and further investigation is required to understand how they govern the differentiation of T_REG_ cells.

### 3.5 The roles of BATF and Irf4 in the generation of TR-T_REG_ cells

During these early differentiating steps, some transcriptional regulators are found to be particularly critical for the generation of TR-T_REG_ over other emT_REG_ subsets. At its core, the acquisition of a tissue residency program of TR-T_REG_ cells is closely matched to the expression of basic leucine zipper ATF-like transcription factor (BATF) and its downstream targets (16). Delacher and colleagues identified a BATF-dependent transcriptional program that drives, notably, the expression of the IL-33 receptor ST2 (120), a receptor specifically found in TR-T_REG_ (17). A T_REG_-specific BATF deficiency in mice (*Foxp3* ^CRE^ *Batf* ^fl/fl^; BATF^-/-^), results in a multiorgan autoimmune disease with death initiating at 6 weeks of age (103). BATF^-/-^ T_REG_ cells fail to accumulate in the lungs, colon, liver, and spleen, and display reduced chromatin accessibility to genes involved in T_REG_ survival in tissue, including *Gata3*, *Irf4, Ikzf4*, *Ets1* and *Icos* (103). In addition, *Foxp3* ^CRE^ *Batf* ^fl/fl^ mice generate exT_REG_ cells that lose T_REG_-associated genes (*Ctla4, Tgfb1, Foxp3*) and adopt inflammatory genes (*Rorc, Il6ra, Stat3*) (103). Specifically, ATAC-seq of murine BATF^WT^ and BATF^-/-^ T_REG_ cells reveals BATF acts as a chromatin regulator, facilitating the expression of TR-T_REG_-associated genes, including *Ctla4*, *Icos*, *Gata3*, and *Irf4*, and preserving the demethylated state of the CNS2 region of *Foxp3* (103), positioning BATF as the epigenetic guardian of T_REG_ cells as they undergo their differentiation into specialized memory T_REG_ cells.

**Figure 2:**
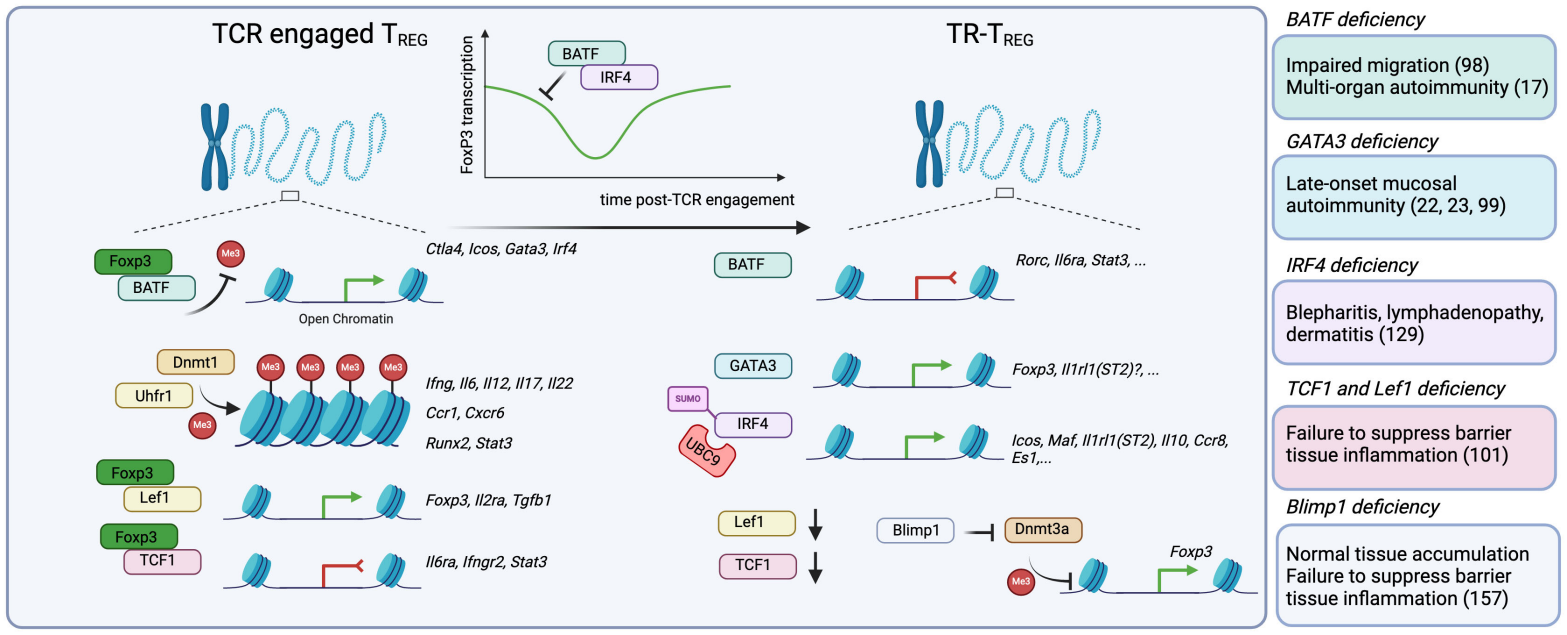
The acquisition of a tissue-resident program requires a series of epigenetic and transcriptional changes that involve modulation of Foxp3 expression or activity. After thymic egress into the periphery, T_REG_ cells are TCR-activated by self or non-self-antigens, and undergo a series of epigenetic and transcriptional changes that guide their maturation into TR-T_REG_ cells. While not entirely understood, this process seems to happen in a step-wise manner. First, TCR-engaged T_REG_ cells upregulate key transcriptional programs in part driven by the transcription factor BATF, which, in conjunction with Foxp3, promotes the accessibility of Foxp3 and expression of BATF-driven genes including *Ctla4, Icos, Gata3, Irf4*. Key to the stability of their epigenetic landscape, T_REG_ cells require Dnmt1 and its partner Uhrf1 to promote the methylation of CpG-rich regions and control the accessibility to inflammatory genes, including *Ifng, Il6, Il12, Il17a, Il22, Ccr1, Cxcr6, Runx2* and *Stat3.* Finally, Foxp3 partners with Lef1 to promote the expression of genes involved in its core program, including *Foxp3, Il2ra* and *Tgfb1*, and also with TCF1 to suppress the expression of genes associated with inflammation like *Il6ra*, *Ifngr2* and *Stat3*. Importantly, BATF and IRF4 can, in turn, suppress Foxp3 expression, a process that, while not fully understood, may enable the temporal accessibility of genes normally repressed by FoxP3. Once in the tissue, BATF enables the continued suppression of genes like *Rorc* (RORγT), *Il6ra* and *Stat3*. GATA3 promotes the transcription of *Foxp3*, but may be further involved in the expression of other GATA3-associated genes, like *Il1rl1(*ST2). IRF4 is also required for the expression of core TR-T_REG_ genes, including *Icos, Il1rl1* and *Il10*. Moreover, there is evidence that *Lef1* and *Tcf7* (TCF1) mRNA expression are significantly decreased in TR-T_REG_ cells, suggesting they are no longer required. Finally, BLIMP-1 expression is increased, and can actively inhibit the action of Dnmt3a, promoting the accessibility of key genes in T_REG_ cells such as *Foxp3*. Consistently, murine models with Foxp3-conditional deletion of BATF, GATA3, IRF4, TCF1 and BLIMP-1 reveal how critical these regulators are for the function of TR-T_REG_ cells.

Another transcription factor (TF) observed to be highly expressed by T_REG_ cells following TCR engagement is the interferon regulatory factor 4 (Irf4) (132). Foxp3 can directly promote the transcription of *Irf4* (133) and the BATF-JUN complex (134). In turn, Irf4 collaborates with BATF to further promote T_REG_ activation, proliferation, and transcriptional differentiation (135). Ding and colleagues demonstrated that upon TCR engagement, T_REG_ cells express the SUMO-conjugating enzyme UBC9 to specifically stabilise Irf4 function (136). While not affecting thymic development of murine T_REG_ cells, a T_REG_-specific deletion of UBC9 causes an early and fatal inflammatory disorder at 3 weeks of age (136), mimicking the dynamics observed in *scurfy* mice (96). These T_REG_ cells show defects in TCR activation, migration, and peripheral accumulation (136). However, we do not observe such dramatic outcomes when knocking out *Irf4* in murine T_REG_ cells, suggesting other factors may compensate for the loss of *Irf4*. Mice harboring a conditional knock-down (*Foxp3* ^CRE^ *Irf4* ^fl/fl^) develop spontaneous dermatitis, blepharitis, and lymphadenopathy disease by 5-6 weeks, and die by 3-4 months from a mostly T_H_2-mediated autoimmune disease (133). Co-immunoprecipitation of Irf4 and Foxp3 shows that both TF interact to, facilitate the transcription of genes such as *Icos*, *Il1rl1*, *Maf* and *Ccr8* (133). In addition, Irf4 allows T_REG_ cells to exert their suppressive functions. For example, a knock-out or a disruption of Irf4 expression in murine or human T_REG_ cells, impacts the expression of key suppressive genes, including *Il10* (137). Moreover, while there is evidence Irf4 is an important contributor during the early transcriptional events involved in the specialisation of activated T_REG_ cells, this TF is also readily detected in some populations of memory T_REG_ cells in the tissue, suggesting its expression is maintained long after TCR engagement. Finally, BATF and Irf4 are particularly upregulated in relation to the strength of the TCR signal (138, 139), and, together, directly suppress *Foxp3* transcription in T_REG_ cells induced *in vitro* (139). Collectively, these observations imply that BATF and Irf4 hinder Foxp3 transcription during the early events that define eT_REG_ formation (**Figure 2**).

## 4 The unique properties of TR-T_REG_ cells

As discussed above, the pool of T_REG_ cells residing in tissues is highly dependent on the organ and is composed in adults of both TR-T_REG_ and emT_REG_ cells whose fate remains ill-defined. Moreover, while the establishment of a peripheral T_REG_ population in mucosal tissues happens in a relatively short amount of time after birth, this is not the case for VAT T_REG_ cells that follow a more gradual accumulation (27), complexifying our understanding of the events that govern TR-T_REG_ accumulation. Notably, fate-mapping systems (*Foxp3* ^eGFPCreERT2^ *x ROSA26* ^STOP-eYFP^) in neonate mice reveal that T_REG_ cells seed non-lymphoid organs like the lungs and liver in the first 8 days of life, persisting for up to 12 weeks with little renewal (25). Critically, exposure to an inflammatory event prior to day 8, but not after, significantly reduces TCR diversity of liver and lung TR-T_REG_ and causes long-lasting alterations to their transcriptional program (25), revealing how critical the neonatal period is to the establishment of tissue homeostasis. Here, the establishment of TR-T_REG_ cells is heavily dependent on the acquisition of a core of transcriptional factors. Single-cell RNA-seq (19), bulk RNA-seq (17), microarray and ATAC-seq (16, 112) analysis of T_REG_ from visceral adipose tissue (VAT), lung, skin or colon reveal the epigenetic and transcriptional landscape of these cells is primarily determined by the organ, with only a small set of core genes shared between them. In various non-lymphoid tissues, TR-T_REG_ cells express a shared a set of core genes, including *Il1rl1* (ST2), *Gata3, Tnfrsf4, Rora*, *Il10* and *Gzmb* (16, 19). On the other hand, there is a significant difference in gene expression between the transcriptional signature and DNA methylation profile of colonic and skin-isolated T_REG_, including increased *Dgat2*, a gene involved in lipid synthesis (16, 19), in skin TR-T_REG_ cells, revealing these cells acquire tissue-specific abilities that allow them to persist in these microenvironments.

### 4.1 Tissue-specific migratory properties of TR-T_REG_

Following TCR engagement and clonal expansion, the development of TR-T_REG_ involves the adoption of migratory properties through the acquisition and loss of chemokine receptors and other adhesion molecules. Indeed, as they undergo deep transcriptional changes and rapid clonal expansion, they also begin to express chemokine receptors that lead them to egress from the lymph node and migrate to a selected tissue. As with other T cells, activated T_REG_ cells downregulate the surface expression of the L-selectin CD62L and upregulate the expression of the glycoprotein type I CD44 (132). Similarly, T_REG_ cells from human tumors (140) and skin (141), as well as murine T_REG_ cells isolated from multiple non-lymphoid organs (15), display low levels of CCR7, preventing their recirculation in lymphoid organs (142). However, the combination of chemokine receptors TR-T_REG_ cells possess is specific to the type of tissue these cells travelled to. In adult mice, RNA sequencing of two distinct populations of T_REG_ cells isolated from barrier tissues reveals that CCR7^-^ T_REG_ possess an organ-specific chemokine receptor signature, regardless of their expression of the IL-33 receptor ST2 (17), suggesting that the migration of all TR-T_REG_ cells is determined by a shared group of chemokine receptors. This combination of chemokine receptors can also be appreciated in the seminal work by Miragaia and colleagues, as they observed skin-localised T_REG_ cells preferentially expressed *Ccr6*, while colonic T_REG_ cells displayed higher levels of *Ccr1* and *Ccr5*; yet, both subsets showed similar levels of *Ccr4*, *Ccr8* and *Ccxr4* (19). Unfortunately, we have yet to determine which combination of chemokine receptors is part of their migratory program and which are locally upregulated to provide further movement inside the tissues.

### 4.2 Core transcription factors of TR-T_REG_ cells

Interestingly, while these experiments highlight the transcriptional diversity of TR-T_REG,_ so did they help identify a core identity that govern their residency program (19). Some members of this list include transcriptional regulators that have been clearly associated to tissue residency in other T cell subsets, like tissue-resident T_RM_ CD8^+^ cells (143, 144), including *Runx3* and *Blimp1* (145). In addition, murine and human TR-T_REG_ also possess unique key markers including transcription factors *Ikzf2, Gata3*, and *Rora*.

#### 4.2.1 Helios

An important transcription factor associated with TR-T_REG_ cells is Helios. While the majority of T_REG_ cells in circulation readily express Helios, siRNA-mediated silencing of Helios expression in human and murine T_REG_ cells does not impede their survival and suppressive capacity *in vitro* (146, 147). On the other hand, the conditional deletion of Helios in murine T_REG_ cells (*Foxp3* ^CRE^ *Ikzf2* ^fl/fl^) leads to the development of a progressive, rather than a *scurfy-*like, lymphoproliferative disease in adult mice (147), revealing it is not required for the development of T_REG_ cells, but rather for the preservation of T_REG_ cell fitness at barrier tissues. Importantly, Helios potentiates the suppressive function of T_REG_ by directly interacting with Foxp3 and promoting histone deacetylation (148), providing further evidence Helios plays a supportive role to the program provided by Foxp3.

However, not all lymphoid and tissue-resident T_REG_ cells express Helios. Originally thought to be solely expressed by tT_REG_ cells (30), it is now well-appreciated that Helios expression in both murine and human Helios^-^ T_REG_ cells is inducible (31, 149) *in vivo* and *in vitro*, respectively. Some of the key features that differentiate splenic Helios^+^ from Helios^-^ T_REG_ is the little overlap they share between their respective TCR repertoire, and the expression of genes involved in the differentiation of specialized T_H_17 cells, including *Rorc*, *Il6ra* and *Il23r* (31), suggesting a division of labor between two T_REG_ subsets that may have long-reaching consequences in the tissue adaptation of TR-T_REG_ cells. For example, Cruz-Morales et al. showed that colonic Helios^+^Gata3^+^ T_REG_ differ greatly from Helios^-^ RORγT^+^ T_REG_ cells by their requirement of CD28, but not MHC-II, to proliferate locally (20), providing a potential point of distinction between colonic Helios^+^ TR-T_REG_ and RORγT^+^ emT_REG_. Nonetheless, further investigation into the role of Helios in the differentiation and maintenance of TR-T_REG_ cells is required.

#### 4.2.2 Gata3

Gata3 is the transcription factor 3 of the Gata-binding family that comprises six known members. In T cells, it has been shown to govern T cell development, proliferation and maintenance (150) and is particularly important to promote the transcriptional signature of helper type 2 T cells (T_H_2) (151). Skin, gastro-intestinal, visceral adipose tissue, and pulmonary TR-T_REG_ cell were all shown to express Gata3 (22, 152), albeit with different intensities. This observation could be explained by the different states of activity of these T_REG_ cells, as Gata3 expression is significantly increased in both murine and human T_REG_ cells upon TCR engagement (22). Interestingly, the signaling pathway that leads T_REG_ to express this TF does not require IL-4 – a cytokine associated with Gata3 expression in conventional T cells (153) – and depends largely on exogenous IL-2 (22). Deletion of Gata3 in murine T_REG_ cells does not lead to the development of spontaneous autoimmunity before 6 months of age (22), after which the mice develop intestinal pathology and dermatitis (104). This is because Gata3-deprived TR-T_REG_ are not hindered in their development, but rather fail to respond to an inflammatory threat, displaying decreased tissue migration, proliferation, transcriptional stability, and suppressive capacity (22, 23, 104).

While not necessary for the maintenance of peripheral tolerance, Gata3 contributes to the functional adaptation of TR-T_REG_ cells. Gata3 recognizes the CNS2 region of *Foxp3* (23), promoting Foxp3 activity and stabilising the transcriptional program of T_REG_ cells to avoid their conversion to pro-inflammatory T cells under stress (22). In addition, Gata3 partners with Foxp3 to form a complex that contributes to the regulation of a wide array of T_REG_-associated genes (104). Gastro-intestinal, skin, pulmonary, and VAT TR-T_REG_ cells express the IL-33 receptor ST2 (17, 24, 154), a known target of Gata3 in T cells (155). Unfortunately, while Gata3 is known to remodel the *Il10* locus in CD4^+^ T cells (156), the link between Gata3 and IL-10 has yet to be established in TR-T_REG_ cells. As such, there are many indicators that Gata3 is an important contributor to the tissue adaptation of T_REG_ cells, and future investigation into the epigenetic, transcriptional, and post-transcriptional impact of this TF is warranted.

#### 4.2.3 RORα

Another gene that is consistently found in RNA-seq data from TR-T_REG_ cells is *Rora*. This gene codes for the retinoic acid receptor-related orphan receptor alpha (RORα), a transcription factor which has been found to be expressed in differentiated T cells, including T_H_1, T_H_2 and T_H_17 (157) cells. Unfortunately, we know very little on the role of RORα in TR-T_REG_. In T cells, *Rora* is expressed upon TCR activation, and is closely associated with the expression of their lineage defining T_H_1, T_H_2 or T_H_17 signature (158). Similarly, RORα plays a supporting role in the transcriptional signature of TR-T_REG_ cells. For example, a Foxp3 conditional deletion of RORα does not alter the accumulation of skin localised TR-T_REG_ cells but enables the evasion of immune responses during skin treatment with MC903, a chemical inducer of atopic dermatitis (18). Thus, as with Gata3, RORα is not required during the transcriptional transformation of tissue-migrating eT_REG_ cells, but rather for their function once in the tissue.

#### 4.2.4 Blimp 1

The B lymphocyte-induced maturation protein-1 (Blimp 1) is a transcriptional regulator that is particularly expressed by T_REG_ cells located in secondary lymphoid organs or non-lymphoid tissues (159). A conditional knock out of *Prdm1* (Blimp-1) in murine T_REG_ (*Foxp3* ^Cre^ *Prdm1* ^fl/fl^) generates an increase in the accumulation of T_REG_ cells, accompanied by small increase in T_CONV_ cell abundance that is insufficient to induce autoimmunity (159), confirming Blimp-1 is not essential to the generation, migration or even function of eT_REG_. Rather, Blimp-1 prevents the methylation of multiple genes, including CNS2 in the *Foxp3* locus, by inhibiting the action of the methyltransferase Dmnt3a downstream of IL-6 (160). In doing so, Blimp-1 prevents the full conversion of colonic T_REG_ to non-suppressive RORγT^+^ eT_REG_ cells (161), suggesting that the role of Blimp-1 is to preserve the transcriptional program of TR-T_REG_ cells.

### 4.3 Tissue-specific survival mechanisms of TR-T_REG_ cells

TR-T_REG_ cells have shown a remarkable capacity to communicate with their immediate environment, adopting cytokine receptors, sensing molecular changes in its environment, and providing direct cell-to-cell contact with immune and non-immune cells (162). TR-T_REG_ achieve this by adopting unique phenotypic characteristics, such as the ability to sense local danger signals and compete in microenvironments with limited IL-2 availability, allowing them to maintain their identity in non-lymphoid organs.

#### 4.3.1 IL-33

IL-33 is a cytokine of the IL-1 family of alarmins constitutively expressed by endothelial and epithelial cells (163) and by activated macrophages and dendritic cells (164). The IL-33 receptor ST2 is transcriptionally upregulated and detected on the surface of TR-T_REG_ (17, 120). This is consistent with the fact that the expression of *Il1rl1* (ST2) is closely associated to the expression of BATF and is part of the transcriptional signature elicited after DNA methylation in TR-T_REG_ cells (16, 120). However, not all tissue isolated T_REG_ express ST2 in mice at the steady state, nor do skin, lung, gut, or VAT-isolated T_REG_ cells express ST2 with the same intensity (17). As such, while suggested as a marker of TR-T_REG_ cells (17), there is currently no clear evidence that ST2 expression is exclusive to TR-T_REG_ cells, and further investigation into this receptor is warranted. Moreover, the importance of ST2 in the differentiation and function of TR-T_REG_ cells remains ill-defined. For example, while IL-33 can directly promote the homeostatic expansion of T_REG_ cells (24, 165), a Foxp3-specific conditional knock-down of ST2 (*Foxp3* ^CRE^ *Il1rl1* ^fl/fl^) does not impair T_REG_ accumulation in the lungs (166). Rather, IL-33 orchestrates T_REG_-mediated suppression of local γδ T (166), T_H_1, and T_H_17 cells during tissue injury (24, 167). To complicate things, it is unclear if these mechanisms depend entirely on the expression of ST2 by T_REG_ cells (168). Indeed, innate immune cells can readily respond to IL-33 and provide proliferative signals to promote TR-T_REG_ expansion and survival (169). As such, rather than providing a survival signal, ST2 may act as a sensing mechanism for local TR-T_REG_ to rapidly reactivate and produce suppressing cytokines.

#### 4.3.2 Icos

While not exclusive to TR-T_REG_, the inducible co-stimulator Icos plays a crucial role in both TR-T_REG_ and emT_REG_ cells to maintain their identity and survival within non-lymphoid organs (21). In mice, a Foxp3 conditional knock out of Icos (*Foxp3* ^YFP-CRE^ *Icos* ^fl/fl^) does not generate autoimmunity, but rather prevents tissue-localised T_REG_ cells from suppressing oxalone-induced dermatitis (170), suggesting Icos is particularly required for T_REG_ cells to control tissue injury. Specifically, Icos coordinates with mTORC1 signaling to support T_REG_ proliferation and the expression of suppressive molecules (171), and is particularly critical for TR-T_REG_ and emT_REG_ cells to persist in the absence of IL-2 signaling by providing anti-apoptotic signals (15). Together, Icos and CD28 act as potent activators of the PI3K/Akt signaling pathway that triggers the phosphorylation of the transcription factor Foxo1 (171, 172). In turn, this sequesters Foxo1 in the cytoplasm and leads to down-regulation of genes like *Klf2* and *Ccr7* (173). In the absence of IL-2, T_REG_ cells become susceptible to apoptosis, highlighting the critical role of sustained Icos-IcosL signaling in their survival as they migrate to the tissue (15). On the other hand, abrogating the PI3K-activating capacity of Icos by removing a YMFM motif in its cytoplasmic tail increases VAT TR-T_REG_ accumulation and function (174), suggesting that Icos may have tissue-specific roles for T_REG_ cells. Thus, while there is abundant evidence that Icos promotes the activation and survival of TR-T_REG_ cells, tissue-specific differences are likely at play and must be considered when investigating TR-T_REG_ cell sub-populations.

### 4.4 The metabolic adaptation of TR-T_REG_ cells

Genes involved in fatty acid β-oxidation (FAO) can be readily detected in antigen-experienced T_REG_ cells isolated from non-lymphoid tissues, including in visceral adipose tissue (VAT), the skin, the colon, and the lungs, suggesting TR-T_REG_ default to FAO in non-inflamed tissues (19, 120). However, these transcriptional approaches have not formally demonstrated that TR-T_REG_ cells require FAO to persist in all tissues. Most of the current evidence comes from VAT-isolated TR-T_REG_, which express the peroxisome proliferator-activated receptor gamma (PPARγ), a ligand-activated transcription factor. Functionally, PPARγ provides a complex signal to engage FAO in VAT T_REG_ cells (175), providing them with a competitive advantage over T_CONV_ cells to survive, accumulate, and function (176). This crucial metabolic strategy enables VAT T_REG_ cells to catabolize long-chain fatty acids (LCFAs) from the environment, turning to FAO to sustain their demand for energy (177, 178). While this process is shared between T_REG_ and T_CONV_ cells, T_REG_ cells utilise fatty acids differently as they do not build endogenous fatty acids from acetyl-CoA, but rely on the acquisition of exogenous fatty acids to meet their metabolic needs (179). Concomitantly, efficient lipid storage by VAT TR-T_REG_ cells is essential to protect them against lipo-toxicity and to provide the metabolic precursors needed for energy generation. These include scavenger proteins such as CD36 and enzymes involved in triglyceride production, such as DGAT1 and DGAT2. Skin and VAT-isolated PPARγ^+^ T_REG_ cells readily express CD36, providing them with the ability to capture and secure LCFAs (175, 180). DGAT are a family of enzymes involved in triglyceride production and lipid droplet (LD) formation that are preferentially expressed in activated T_REG_ cells (181). Foxp3 itself is a strong repressor of *Glut1 (* 182), the glucose transporter, and favors the expression of FAO genes (178). Yet, this mechanism acts in a feed-back loop, with DGAT1 promoting Foxp3 expression by diminishing protein kinase C (PKC) activity downstream of the TCR (181, 183). Interestingly, by tracing the tissue distribution of splenic T_REG_ cells with shared TCR sequences, Li et al. demonstrated that PPARγ-expressing eT_REG_ cells localise to other non-lymphoid sites, including the skin and the liver (184), providing new translational evidence that FAO proteins are expressed by other TR-T_REG_ cells. Nonetheless, while these observations highlight the importance of FAO for VAT TR-T_REG_ cells to sustain their bioenergetic demands, it remains to be determined if this metabolic strategy is required to sustain other TR-T_REG_ cells.

## 5 The inflammatory adaptation of TR-T_REG_

**Figure 3:**
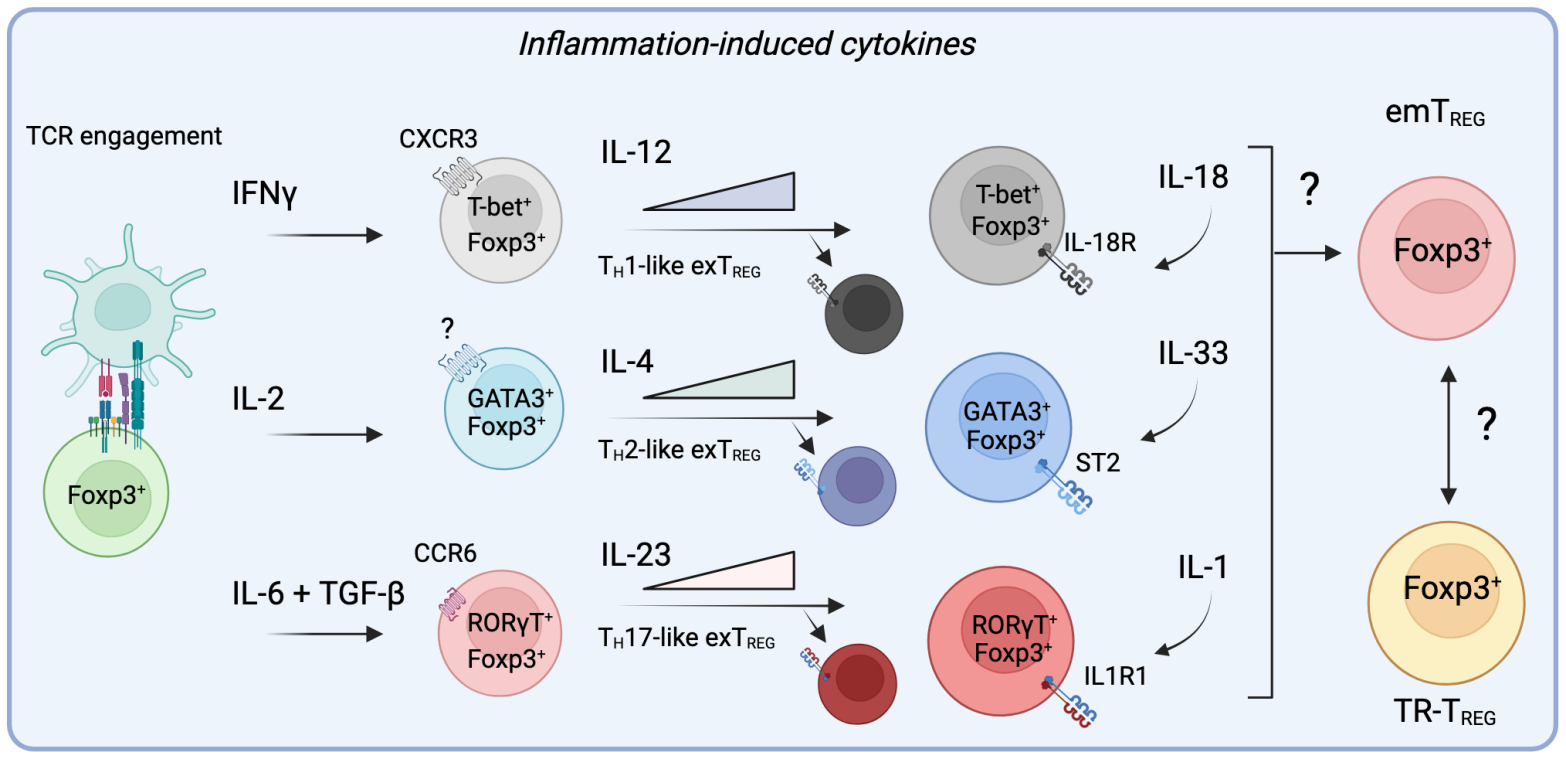
Specific inflammatory signals alter the trajectory of T_REG_ cells in non-lymphoid sites by engaging specialized programs prior and during their migration to inflamed tissues. During active inflammation, the presence of cytokines such as IFNγ, IL-2, IL-6, and TGF-β can divert the differentiation of T_REG_ cells to adopt helper-like phenotypes, allowing them to migrate to specific sites of inflammation alongside conventional T cells. Importantly, by acquiring these master transcription factors, effector T_REG_ cells (eT_REG_) become responsive to signals provided by IL-12, IL-4 or IL-23. While these cytokines further promote the transcriptional program engaged by these specialized T_REG_ cells, they can ultimately diminish their suppressive functions and allow them them to contribute to inflammation as exT_REG_ cells. Importantly, it remains to be determined if the resulting population of emT_REG_ cells in the tissue after inflammation acquire a residency program that lead them to form part of the TR-T_REG_ cell population.

One of the most recent and exciting discoveries has been the observation that activated eT_REG_ can further specialize to adopt T_H_1, T_H_2, T_H_17, and even T_FH_-like features. Importantly, they can express master transcription factors that are part of a transcriptional program typically expressed by helper T cells, including T-bet (T_H_1), RORγT(T_H_17), Gata3(T_H_2), and BCL6 (T_FH_). The differentiation, migration, and tissue accumulation of functionally-specialized T_REG_ cells in tissues is a dynamic process that can occur in microbiota-rich barrier sites (10) or during tissue injury (185, 186). Indeed, contrary to the core genes necessary for the generation and maintenance of TR-T_REG_ cells, the role of these “master” transcription factors is not associated with a residency program; rather, these TFs promote a set of specialized functions that allow T_REG_ cells to suppress or orchestrate local immune responses (**Figure 3**). For example, single-cell analysis performed at distinct times during an Influenza A infection in mice portrays how Gata3^+^ T_REG_ cells are progressively replaced by antigen-specific T-bet^+^CXCR3^+^ T_REG_ cells in the course of disease, suggesting that, contrary to the permanent presence of TR-T_REG_, T_H_1-specialized T_REG_ cells are generated concurrently with the antiviral T_H_1 response and follow the pattern of accumulation of these cells (185, 187).

Interestingly, some of these specialized T_REG_ cells (RORγT^+^ T_REG_) are present at the steady-state in mucosal tissues such as the colon, blurring attempts at defining what constitutes the *bona fide* TR-T_REG_ phenotype in these tissues. Indeed, key events leading to the generation of specialized T_REG_ cells include the requirement for TCR signaling and aerobic glycolysis to facilitate clonal expansion and differentiation (188). Moreover, Irf4 (27) is a necessary stepping-stone for the differentiation of specialized T_REG_ cells (9, 28, 29). A typical example of these specialized T_REG_ cells is observed in the colon, where resident T_REG_ cells displaying two distinct TCR repertoires can be segregated based on their transcriptional program. Indeed, both RORγT^+^ T_REG_ and Gata3^+^ T_REG_ are readily detected in the colon; however, absence of a local microflora only hinders the specific generation of RORγT^+^ T_REG_ (189, 190) since their TCR repertoire is largely biased towards bacterial antigens (72, 191, 192). Since specific signals are required for T_REG_ cells to acquire these programs, it is possible to dissect the required pathways that lead T_REG_ cells to acquire these specialized programs.

### 5.1 The effects of polarizing signals on the fate of T_REG_ cells

Some of the better described signals that promote the generation of specialized T_REG_ cells include cytokines that drive the phosphorylation and nuclear translocation of STAT and SMAD proteins (193). In turn, these signals promote the expression of genes that define T cell fate, including the acquisition of master transcription factors T-bet, Gata3, or RORγT. What is particularly interesting, however, is that the pathways that lead T_REG_ to adopt these TFs can also undermine their Foxp3-dependent transcriptional program, either through the loss of Foxp3 expression, the expression of pro-inflammatory genes, or the engagement of apoptosis. As such, at the time when activated T_REG_ cells undergo important epigenetic and transcriptional changes, certain inflammatory signals can promote the loss of Foxp3 expression and their conversion into inflammatory “exT_REG_” cells. Several key transcription factors have been described to be involved in this inflammatory adaptation process of T_REG_ cells.

#### 5.1.1 T-bet^+^ eT_REG_

T-bet is a T-box transcription factor expressed in a wide variety of immune cells, and mostly recognized for its role in defining the transcriptional landscape of T_H_1 cells (194). Using a unique murine model that enables the tracking of murine T-bet-expressing T_REG_ (*Foxp3* ^Thy1.1^ *Tbx21* ^tdTomato-T2A-CreERT2^ *R26YFP* ^-fl-stop-fl^), Levine and colleagues showed that the conditional deletion of T-bet in Foxp3^+^ T_REG_ cells does not lead to autoimmunity in adult mice, although it does generate a mild increase in T_H_1 activity (195), suggesting T-bet has little to no impact on the way T_REG_ preserve tissue function at the steady-state. Notably, T-bet is a critical regulator for the expression of CXCR3 (196), a chemokine receptor that orchestrates eT_REG_ migration to sites of T_H_1-driven inflammation (196, 197). Highlighting the role of TCR engagement, T-bet^+^ eT_REG_ cells that progressively accumulate in the lungs of mice infected with acute Influenza A infection recognize viral proteins (185, 198). Thus, as with T_H_1 cell polarization, the generation of T-bet^+^ eT_REG_ occurs progressively during inflammation and is closely associated to the clonal expansion of antigen-specific CD4^+^ T_H_1 cells.

The signals that promote the generation of T_H_1 cells include IFNγ (STAT1) and IL-12 (STAT4). Interestingly, an IFNγ-STAT1 signal drives the initial expression of T-bet during TCR engagement, while a subsequent IL-12-STAT4 signal is required for their definitive differentiation (199, 200). This initial T-bet expression can, in turn, promote the expression of the IL-12 receptor (IL-12Rβ2) (201–203). However, contrary to T_H_1 cells, eT_REG_ cells seem to depend exclusively on the presence of IFNγ for the acquisition of T-bet (196, 204). By activating murine CD4^+^Foxp3^+^ cells *in vitro*, Koch and colleagues demonstrated that T_REG_ cells acquire T-bet expression and its associated target, CXCR3, only if they possess the receptor IFNγR1 (205), suggesting that IFNγ-producing T_H_1 cells are responsible for the polarization of T_H_1-like eT_REG_ cells.

The control of IL-12 signalling by T_REG_ cells is critical, as excessive pSTAT4 can lead T_REG_ cells to lose Foxp3 expression (206) by, notably, limiting chromatin accessibility of STAT5 to the *Foxp3* locus (207). Yet, STAT4 is a major regulator of *Ifng* in CD4^+^ T cells (208), and both human and murine T_REG_ exposed to IL-12 produce low levels of IFNγ (187, 205, 206, 209–212), revealing excessive IL-12 can still be perceived by T_H_1-like eT_REG_ cells. However, contrary to STAT1, STAT4 signaling is associated with less suppressive T_REG_ cells and can even lead to the complete loss of Foxp3 expression (187, 205, 206, 209–211), suggesting T-bet^+^ eT_REG_ are in a constant struggle to avoid the loss of genes involved in their suppressive functions. In this regard, T-bet^+^ T_REG_ cells possess mechanisms to avoid overt STAT4 signaling. For example, IFNγ-induced T-bet^+^ eT_REG_ cells suppress IL-12Rβ2 surface expression, preventing excessive phosphorylation of STAT4 and further T_H_1-like commitment (205). Moreover, non-labelled proteomics on circulating human T_REG_ cell populations revealed that, compared to memory or naïve T_REG_, eT_REG_ maintain low cytosolic levels of STAT4 (213).

There is growing evidence for the role of IL-18 on the function of tissue-resident T-bet^+^ eT_REG_ cells. While the origin of IL-18R1^+^ eT_REG_ cell remain to be fully understood, T_H_1 polarizing conditions, and particularly IL-12, allow T_REG_ cells to adopt the expression of both T-bet and IL-18R1 (187), suggesting that, like for T_conv_ cells, eT_REG_ require a STAT4-dependent chromatin remodeling to express IL-18R1 (214, 215). *In vitro*, IL-18 promotes the expansion and suppressive capacity of IL-12-generated T-bet^+^ T_REG_ cells (187), suggesting this signal can counter the destabilising effects of IL-12. *In vivo*, T-bet^+^ eT_REG_ cells express IL-18R1 when they accumulate in the lungs during an Influenza A infection (187). Here, IL-18 enhances the production of amphiregulin in local T_REG_ cells, facilitating tissue restoration after pulmonary Influenza A infection (216). In addition, a Foxp3 conditional knock-out of *Il18r1* (*Foxp3* ^ERT2-CRE^ *Il18r1* ^fl/fl^) allowed us to demonstrate that IL-18 is specifically required for eT_REG_ cells to suppress IL-17A responses in the lungs after an Influenza A infection (187). Similarly, IL-18R1 deficiency in T_REG_ cells fails to control the onset of a T cell-mediated colitis (217) as well as inflammation in an experimental model of ovalbumin-induced asthma (218), confirming IL-18 is an important contributor to eT_REG_ function. However, these observations do not necessarily mean that IL-18R1 expression is restricted to T-bet^+^ T_REG_, as we have observed RORγT expression among a subset of IL-18R1^+^ T_REG_ cells (187) and IL-18R1 expression has been described in T_H_17 cells (217). Collectively, these observations illustrate how the T_H_1 adaptation of eT_REG_ cells allows for the suppression of tissue inflammation.

#### 5.1.2 Gata3^+^ eT_REG_

The transcription factor Gata3, which is an important component of the transcriptional program of TR-T_REG_, is best described for its role in driving T_H_2 cell differentiation (219). In both human and murine CD4^+^ T cells, Gata3 promotes T_H_2-associated genes, allowing for the expression of genes associated to their function, such as IL-4, IL-5, and IL-13 (151, 219). There are numerous accounts of tissue-homing T_REG_ cells expressing high levels of Gata3 during acute T_H_2-driven immunity, such as what is observed during asthma (220) or helminth infections (221, 222).

The signals driving GATA-3 expression in T_REG_ cells are not fully understood. Two signals have been described to be sufficient to induce Gata3 expression during T_H_2 differentiation, namely an IL2/STAT5-dependent and an IL-4/STAT6-dependent signal (223–225). In homeostatic conditions, IL-2 (STAT5) is sufficient to promote the expression of Gata3 during TCR engagement (22). However, in T_H_2-driven responses, T_REG_ cells require IL-4R to acquire GATA-3 expression and their T_H_2-like characteristics (226). This distinction between STAT5 and STAT6-dependent induction of Gata3 may pave the way towards understanding how T_H_2-like eT_REG_ cells differ from TR-T_REG_ cells. For example, mice with a Foxp3-specific conditional knock-down of *Il4ra* (*Foxp3* ^CRE^ *Il4ra* ^fl/fl^) fail to prevent exacerbated asthma-like symptoms when challenged with house-dust-mite (HDM) (226) and helminth-driven inflammation, despite the presence of T_REG_ cells *in situ* (221).

While IL-4 can favor T_REG_ cell-mediated functions, sustained IL-4 can also force T_REG_ cells to lose Foxp3 expression and their suppressive capacity both *in vitro* (227) and *in vivo* (221, 222, 227). STAT6 can promote the activity of the histone deacetylase HDAC9, which decreases chromatin accessibility to the *Foxp3* locus (228). To prevent this, eT_REG_ cells require strategies to avoid excessive IL-4 signaling. First, by maintaining high levels of CD25 expression, eT_REG_ cells remain sensitive to IL-2, whose STAT5 signal competes with STAT6 activity (229). Second, tissue-localised T_REG_ cells prevent further commitment into the T_H_2 lineage by producing the E3 ubiquitin ligase Itch (230, 231). Finally, murine *in vitro*-induced T_REG_ cells exposed to IL-4 express higher levels of the JAK/STAT inhibitor SOCS2 to prevent further STAT6 phosphorylation and the expression of pro-inflammatory cytokines (232). Thus, while it remains to be fully confirmed in tissue-resident T_REG_ cells, there is cumulating evidence that IL-4 is important for the commitment of Gata3^+^ eT_REG_ cells, and responsible for their transcriptional destabilisation and conversion into T_H_2-like ex-T_REG_ cells.

Finally, IL-33, which contributes to the proliferation of TR-T_REG_ cells (165), can also govern the function of Gata3^+^ eT_REG_ cells during inflammation. In this regard, IL-33-responding activated T_REG_ cells where shown to produce high amounts of IL-10 and TGF-β (233), playing a key role in maintaining intestinal homeostasis (24). Similarly, ST2^+^ T_REG_ cells promote the suppression of anti-tumor immune responses (234–236). However, IL-33 can also drive the production of the T_H_2 associated cytokines IL-5 and IL-13 in pulmonary eT_REG_ cells (233, 237, 238) and interfere with their capacity to supress T_H_2 responses (238). Thus, the role of IL-33 on Gata3^+^ T_REG_ cells is specific to the inflammatory context and may depend on whether it targets TR-T_REG_ cells or eT_REG_ cells accompanying T_H_2 responses.

#### 5.1.3 RORγT^+^ eT_REG_

While complex and not entirely defined, the signaling events that lead T_REG_ cells to adopt a T_H_17-like phenotype include some of the same polarizing JAK-STAT and SMAD signals that are required for the generation of T_H_17 cells. Indeed, the promoter functions of both Stat3 (239) and RORγT (240) are required to establish a T_H_17 cell transcriptional program (241), and T_REG_ cells have been shown to share part of this transcriptional program through the acquisition of these TFs (239). In the gut, RORγT^+^ T_REG_ cells play an essential part in maintaining gut homeostasis and contribute to maintain local homeostasis by, notably, suppressing T_H_17-driven responses (242). Transcriptionally, RORγT^+^ T_REG_ cells from the mouse colon at steady-state express higher levels of *Il23r, Il1r1, Maf, Irf4*, and *Ikzf3* than their RORγT^-^ counterparts (191), revealing they possess a unique landscape that encompasses some key T_H_17-associated genes. Moreover, RORγT is required for IL-10 production by colonic T_REG_ cells and prevention of T cell-mediated colitis (191). Similarly, RORγT is required for T_REG_ cells to control T_H_17-mediated autoimmune arthritis and EAE (192, 243), suggesting that RORγT expression allows emT_REG_ cells to target and suppress T_H_17-driven responses. However, the role of RORγT and its impact on the transcriptional landscape of emT_REG_ cells remains ill-defined and is likely driven by the inflammatory microenvironments these cells are exposed to.

While many cytokines can promote the nuclear translocation of Stat3 in T_H_17 cells, the simultaneous signals provided by TGF-β (SMAD2/3) and IL-6 (Stat3) are sufficient, *in vitro*, to induce RORγT expression in T_REG_ cells (162, 192). Interestingly, a delicate balance is achieved between the signal provided by TGF-β and IL-6. For example, TGF-β and IL-6 synergistically promote the proteasome-dependent degradation pathway of Foxp3 (244), contributing to a partial loss of Foxp3 function. Interestingly, once colonic RORγT^+^ T_REG_ cells are generated, they display a significantly stable phenotype, with maintained demethylation of T_REG_-specific genes like *Foxp3, Ikzf2, Ctla4, Gitr* and *Ikzf4* (Eos) (191). In fact, these cells possess intrinsic mechanisms to avoid their full conversion towards T_H_17 cells. As with IL-12 and T_H_1 cells, subsequent signals provided by IL-23(Stat3) can further destabilise the transcriptional program of RORγT^+^ T_REG_ cells and even engage an apoptotic cascade in these cells (245). Indeed, *Il23r* is amongst the genes upregulated by Stat3 and RORγT (246), making RORγT^+^ eT_REG_ particularly sensitive to IL-23 (245). In a recent report, Jacobse and colleagues demonstrated IL-23R expression is restricted to RORγT^+^ T_REG_ under homeostatic conditions in the colon, and colonic T_REG_ cells maintain a competitive advantage over WT T_REG_ cells to survive in these conditions (245). Concomitantly, the authors demonstrate that T_REG_ cells isolated from the lamina propria of patients with active IBD express high levels of *Il23r* and pro-apoptotic genes (126), suggesting an evolutionary conserved mechanism that orchestrates RORγT^+^ eT_REG_ survival and function.

In addition to IL-23, IL-1β was found to promote the differentiation of human CD4^+^CD25^high^CD127^low^ Foxp3^+^ T_REG_ cells into IL-17-producing cells (247, 248), suggesting IL-1 may promote a pro-inflammatory phenotype in T_REG_ cells. However, the role of IL-1 in RORγT^+^ eT_REG_ cells remains ill-defined. Through a T-cell mediated colitis model in mice, we demonstrated that a knock-out of IL-1R1 in T_REG_ cells favors an accumulation of Gata3^+^ T_REG_ cells over RORγT^+^ T_REG_ cells in the colon, as IL-1 directly promotes RORγT^+^ T_REG_ expansion (167). Despite this effect, a lack of IL-1 signaling in T_REG_ cells results in more abundant accumulation in the colon compared to WT T_REG_ (167), suggesting IL-1 is a negative signal for the maintenance of colonic T_REG_ cells. Interestingly, there are specific situations where this effect is beneficial. For example, IL-1R1^-/-^ mice infected with *Cryptococcus neoformans* are particularly sensitive to the infection, as they cannot mount an effective T cell response (249). In their lungs, these mice lack RORγT^+^ T_REG_ cells and have increased ST2^+^ T_REG_ cells in the lungs compared to WT mice (167), suggesting sustained immunosuppression. To counter this, activated T_REG_ cells express high levels of the decoy receptor IL-1R2, which allows them to neutralize IL-1 signalling (250–252).

## 6 Conclusion

In this review, we aimed to detail some of the major elements that govern the trajectory of a precursor T_REG_P cell to a highly specialized TR-T_REG_ cell. It is particularly interesting that the trajectory of a T_REG_ cells is, in most regards, highly like that of the conventional T cell as it undergoes further polarization prior to reaching peripheral tissues. Importantly, the epigenetic malleability of T_REG_ cells is central to their ability to perform outside of the thymus, as these transformations allow them to sense tissue-derived signals that, in turn, modulate their suppressive functions. However, while we have accumulated a lot of information in recent years, much remains to be understood on how these tissue and inflammation-specific adaptations govern the function of TR-T_REG_ cells. For example, the notion that T_REG_ cells can adopt a specific differentiation path and revert to their previous state, labelled “plasticity” (253, 254), remains to be proven experimentally.

Finally, recent reviews have addressed how *Foxp3* gene editing, IL-2 therapy, and the use of T_REG_ cells as cellular therapies represent key strategies to engage human T_REG_ cells (255). However, most of our current knowledge on TR-T_REG_ cells has not been specifically exploited by T_REG_-targeting therapeutical approaches. There is, however, some evidence these strategies may facilitate the expression of a tissue residency program. For example, the development of muteins or low-dose therapies (256) aimed at promoting IL-2 signaling in T_REG_ cells can promote the expression of genes associated with TR-T_REG_ cell function, such as *Il1rl1*(ST2), as well as migratory and other tissue resident genes (257). Thus, it is of interest to understand how T_REG_ targeting strategies can influence both the developmental trajectory and the function of tissue resident T_REG_ cells. In addition, understanding the migratory cues that enable TR-T_REG_ cells to recognize specific tissues can have long reaching therapeutical benefits. Chimeric antigen receptor (CAR) T_REG_ cells have been proposed as a new avenue to circumvent the constraints of low T_REG_ cells numbers and the unknown TCR repertoire of T_REG_ in autoimmune or graft-versus-host (GvHD) diseases (258). However, this approach is still very novel, and, in the absence of additional modifications, is expected to suffer from the same limitations of CAR-T cells (258, 259), including failing to adopt metabolic strategies to survive, preventing exhaustion, and maintaining their function in tissues. Thus, it is by establishing a solid understanding of the entire pathway leading T_REG_ cells to adapt to non-lymphoid organs that we provide the basis for the development of better T_REG_ cell-based therapies.

## Author contributions

FA: Writing – original draft, Writing – review & editing. ZL: Writing – original draft, Writing – review & editing. AB: Writing – original draft, Writing – review & editing. CP: Writing – original draft, Writing – review & editing, Conceptualization, Funding acquisition, Project administration, Supervision.
